# Quantification of the Dental Morphology of Orangutans

**DOI:** 10.1155/2013/213757

**Published:** 2013-11-18

**Authors:** P. Nambiar, J. John, Samah M. Al-Amery, K. Purmal, W. L. Chai, W. C. Ngeow, N. H. Mohamed, S. Vellayan

**Affiliations:** ^1^Department of Diagnostic and Integrated Dental Practice, Faculty of Dentistry, University of Malaya, 50603 Kuala Lumpur, Malaysia; ^2^School of Dental Sciences, Universiti Sains Malaysia, 16150 Kubang Kerian, Kelantan, Malaysia; ^3^Department of Oral and Maxillofacial Surgery, Faculty of Dentistry, University of Malaya, 50603 Kuala Lumpur, Malaysia; ^4^Faculty of Pharmacy, Universiti Teknologi MARA, Puncak Alam, 42300 Kuala Selangor, Selangor, Malaysia

## Abstract

Orangutans are believed to have close biological affinities to humans. Teeth being the hardest tissue provide useful information on primate evolution. Furthermore, knowledge of the pulp chamber and root canal morphology is important for dental treatment. A female Bornean orangutan and a Sumatran male orangutan skull were available for this study. Both of their dentitions, comprising 50 teeth, were scanned employing the cone-beam computed tomography for both metrical and nonmetrical analyses. Measurements included tooth and crown length, root length, enamel covered crown height, root canal length (posterior teeth), length of pulpal space (anterior teeth), and root canal width. Nonmetrical parameters included number of canals per root, number of foramina in each root, and root canal morphology according to Vertucci's classification. It was found that the enamel covered crown height was the longest in the upper central incisors although the canine was the longest amongst the anterior teeth. Both the upper premolars were three-rooted while the lower second premolar of the Sumatran orangutan was two-rooted, with two foramina. The mandibular lateral incisors of the Bornean orangutan were longer than the central incisors, a feature similar to humans. In addition, secondary dentine deposition was noticed, a feature consistent with aged humans.

## 1. Introduction

Orangutans (*Pongo *spp.), the Southeast Asian great apes, are interesting primates considered to be the closest living relative to human beings. Orangutans have a minimum of 28 distinctive physical characteristics similar to humans but only two characteristics with chimpanzees and seven with gorillas [1]. Most researchers recognize two separate subspecies, *Pongo abelii* in Sumatra and *Pongo pygmaeus* in Borneo. Current studies prove that the two taxonomic groups are geographically isolated for 10,000–15,000 years [2].

Several previous studies [2, 3] have listed some general external characteristics to differentiate the two species. Bornean orangutan has been described as having hair colour ranging from red to deep maroon or blackish brown. In contrast, Sumatran orangutan's hair colour is lighter, rusty red, or light cinnamon. However, there is an extreme form of Sumatran orangutan which has dark-haired colour similar to the Bornean [4], indicating the need for caution in the use of colour as a taxonomic marker. In addition, the variation in the dental morphology and cranial measurements within Bornean orangutan is greater than or equal to the variation between Bornean and Sumatran orangutan [5]. Comparable differences in the cranial measurements were also observed within the Bornean population [6].

The variations between the Bornean and Sumatran may be ontogenetic responses to different environments they reside in [4]. Several researchers have established the relationship between dental enamel thickness and their diet. Hard-object feeders have thicker enamel than soft-object feeders [7]. The Bornean [8] showed strong mandibles with high resistance to teeth fractures during the mastication process. This difference in the strength of mandible was due to the nature of the food consumed by the different species. This theory is supported by the finding [9] that Bornean orangutan often consumes more tough food than the Sumatran orangutan, which feeds more on ripe fruit available throughout the year. Although the male orangutan consumes harder food, both Bornean and Sumatran females have been noted to consume more bark than males [10].

The enamel thickness indices between orangutan maxillary and mandibular teeth show similar pattern to other great apes and humans [11] with the average thickness increasing from the anterior to the posterior teeth. However, orangutan shows unusual enamel thickness in their central incisors. This thickness is related to bark gouging or incisal biting of the mechanically demanding foods [8]. The maxillary molars tend to be broader in a buccolingual dimension compared to the mandibular molars, providing it with the wide cross-sectional area required [12]. Both orangutan and human dentition are well known to be dimorphic, where the males' exhibit larger dentine cores and therefore have larger teeth [13]. In addition, the orangutan male exhibits greater or equal enamel cap area value compared to the female [14].

Tooth morphology information is useful for understanding human and primate evolution. As teeth are the most calcified tissue, fossilized teeth have durable postmortem preservation and have been used to study the evolutionary linkage of human and other primates. It is also reported [15] that signs of dental diseases in primates can be misleading as the proximity of the dentition to many important structures may cause bacterial infections of the inner ear, sinuses, and possibly the eyes. Moreover, knowledge of root canal morphology is important for both surgical and nonsurgical endodontic therapies. A cone-beam computed tomography (CBCT) machine is a perfect scanning machine to determine the root canal system in different planes simultaneously [16]. Recognition of variations in root canal anatomy is an essential prerequisite for successful endodontic diagnosis and treatment. Besides the main advantage of low radiation dosage when compared to the cumbersome medical multidetector/helical CT, CBCT is capable of measuring dental structures in different direction wherein the images are undistorted and exact in size [17].

At present little is known about root canal morphology and size of orangutan teeth. Two of the authors (Purmal and Nambiar) successfully treated a Bornean orangutan's carious canine tooth [18], possibly caused by an unusual defective enamel development. This research was therefore undertaken to understand the dental anatomy and pulpal morphology of the two different orangutans, *Pongo abelii* and *Pongo pygmaeus*, and to make some comparisons to human dentition.

## 2. Materials and Methods

### 2.1. Imaging with CBCT

**Figure 1:**
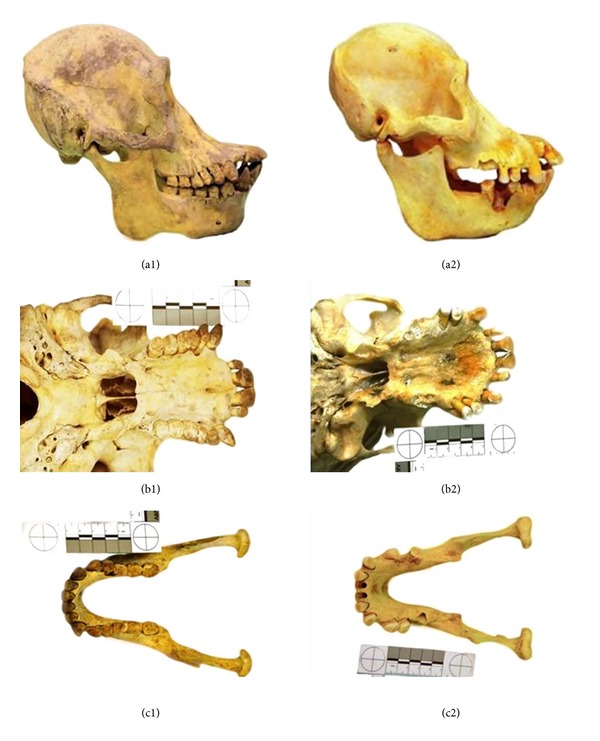
(a1) Bornean orangutan skull; (b1) Bornean orangutan maxillary arch; (c1) Bornean orangutan mandible arch; (a2) Sumatran orangutan skull; (b2) Sumatran orangutan maxillary arch; (c2) Sumatran orangutan mandibular arch.

Two well-preserved skulls were obtained with permission from the National Zoo, located at Kuala Lumpur, Malaysia. One was a Bornean female with near full dentition (Figure 1(a)) while the other was a Sumatran male with a partial dentition (Figure 1(b)). Scanning of the dentition was performed employing the Kodak 9000 3D CBCT extraoral imaging system (Carestream Health, Inc.) at the Oral and Maxillofacial Imaging Division, Faculty of Dentistry, University of Malaya. This machine recorded the scanned volume at a field-of-view (FOV) size of 5 × 3.8 cm with a spatial resolution of 76 mm and grey resolution of 14 bits. An advantage of this CBCT machine is that the images can be studied by using different representations (multiplanar reformation, and 3D surface rendering). These various images were reconstructed from the CBCT data using the proprietary Kodak Dental Imaging Software 3D module version 3.1.9. This study examined 50 teeth obtained from imaging the two orangutans' skulls, thirty-two in the Bornean orangutan and eighteen in the Sumatran orangutan (Table 1).

### 2.2. Metrical Measurements

**Figure 2:**
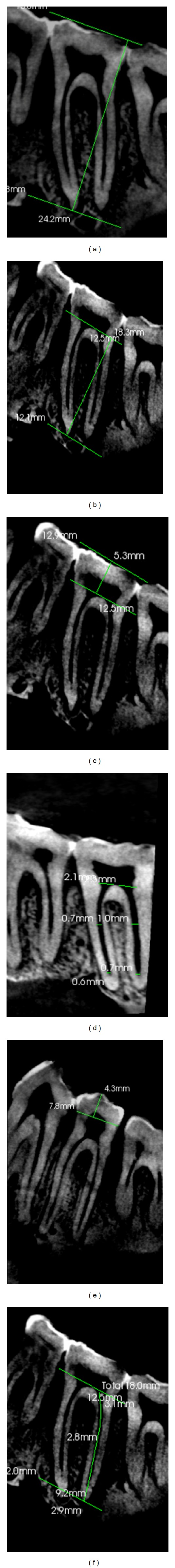
(a) Total tooth length; (b) root length; (c) crown length; (d) root canal width; (e) enamel covered crown length; (f) root canal length.

Several measurements (Figure 2) were performed to study the size of orangutan teeth and pulpal canal. A method of measuring the teeth needed to be devised as the orangutan teeth were different from human. In human, the crown was the enamel covered portion while the length of the root could be measured from the cement-enamel junction to the apex of the roots. As the orangutan had an excessive length on the mesial portion of its tooth compared to the distal, a definite method of measurement was required. Tooth length (Figure 2(a)) was measured from occlusal plane surface (highest cusp tip tips as the reference point points) to the apical foramen of the longest root. Both the crown height and its related root length were measured after a line was drawn at the level of the bi/trifurcating roots in the posterior teeth. This line was drawn parallel to the occlusal plane of the particular tooth. The root length (Figure 2(b)) was measured as the distance from this line to the apex of the root. The crown length (Figure 2(c)) was measured as the distance from the occlusal surface to the line at the level of the bifurcation area. Root canal width (Figure 2(d)) was measured at three levels: (i) at the level of the root canal orifice; (ii) visible canal at the root apex; and (iii) at the midpoint between root canal orifice and the visible canal at the root apex. Additional measurement was performed to determine the area covered by enamel (Figure 2(e)). This was measured as the distance from the occlusal cuspal tip to the level of the endpoint of the enamel covered crown. The actual root canal length (Figure 2(f)) was measured from the level of the root canal orifice to apical foramen, following closely the curvature of the root canal. The anterior teeth are all single-rooted. Both the tooth length and the enamel covered area were measured following the same method as that for the posterior teeth. Crown length, root length, and root canal length were omitted because of the absence of the bifurcation of the roots. The length of the pulp space was measured as a distance from the maximum point of pulp chamber occlusally to the maximum point of the visible root canal apically.

### 2.3. Nonmetrical Observations

**Figure 3:**
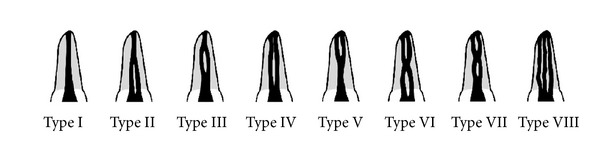
Vertucci's classification of root canal morphology adapted from Vertucci [26].

Additional investigations were conducted on the axial images, especially the number of the apical foramina for each root and number of canals in each root. This view was also important in confirming the root canal morphology according to Vertucci's classification (Figure 3). Data was entered and analyzed using SPSS 12.01 for windows (SPSS Inc., Chicago, USA).

## 3. Results

Tables 2 and 3 summarize the results of the various metrical measurements in this study. It was noticed that the height of the crown covered by enamel in the maxillary anterior teeth was larger in the Bornean orangutan. It ranged between 7.1 and 13.8 mm, while in the Sumatran's skull it ranged between 8.1 and 11.3 mm. An interesting finding is that the enamel covered crown height (ECCH) of the central incisors was the longest despite the canines being the longest anterior teeth. In contrast, this feature was not observed on the lower anterior teeth. The longest ECCH was found on the mandibular caninet, which was also the longest mandibular anterior tooth. In addition it was observed that the Sumatran orangutan had longer maxillary canines than the Bornean female, with a difference of 5.8 mm and 4.1 mm for the right and left canines, respectively. The mandibular canines did not show any significant differences between the two species (Table 2).

According to the measurements of the posterior teeth, the maxillary first premolar was longer in the Bornean skull than in the Sumatran skull with a length of 28.3 mm and 29.1 mm for right and left side, respectively, while the corresponding teeth of the Sumatran male measured 26 mm and 25.9 mm. Unlike humans, the orangutans had unique premolars as they were three-rooted. The palatal roots were longer than the other roots, which were the mesiobuccal and distobuccal roots. The maxillary molar root system also demonstrated a similar pattern. The lower dentition exhibited mesial roots longer than the distal roots; this variation in the root length was clearly seen in the Sumatran orangutan with a range of 17.7–23.8 mm for the mesial root and 16.9–20.5 mm for distal root (Table 3).

**Figure 4:**
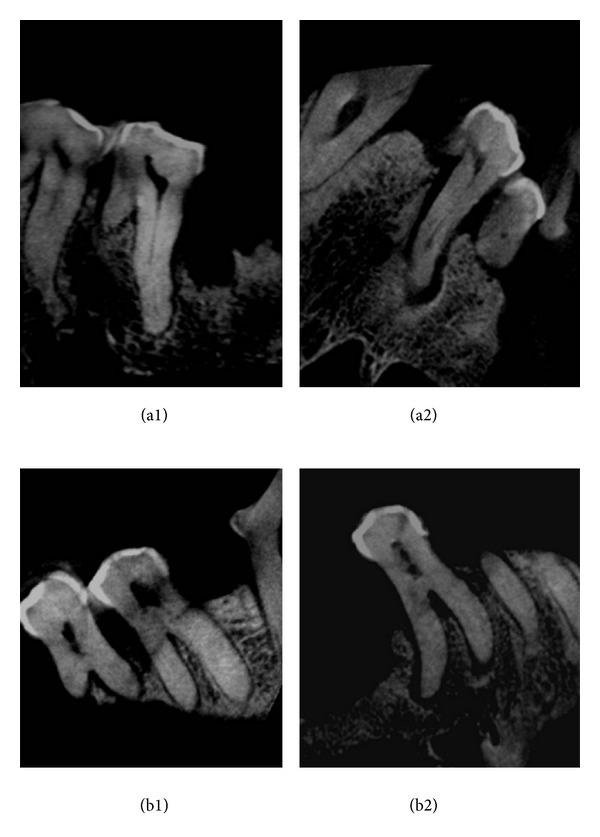
(a1 and a2) Partially calcified teeth; (b1 and b2) fully calcified or completely obliterated teeth.

Table 4 shows that the maximum width of the root canal was at the level of the root canal orifice of each root. Subsequently, the canal was tapering towards the apex. Interestingly the palatal root canal width was the widest as well as the longest. The Sumatran male was probably the older between the two as it had more missing teeth as well as partially obliterated root canals. The pattern of calcification looks similar to humans, whereby the pulp chamber and root canal become reduced as age advances (Figure 4).

**Figure 5:**
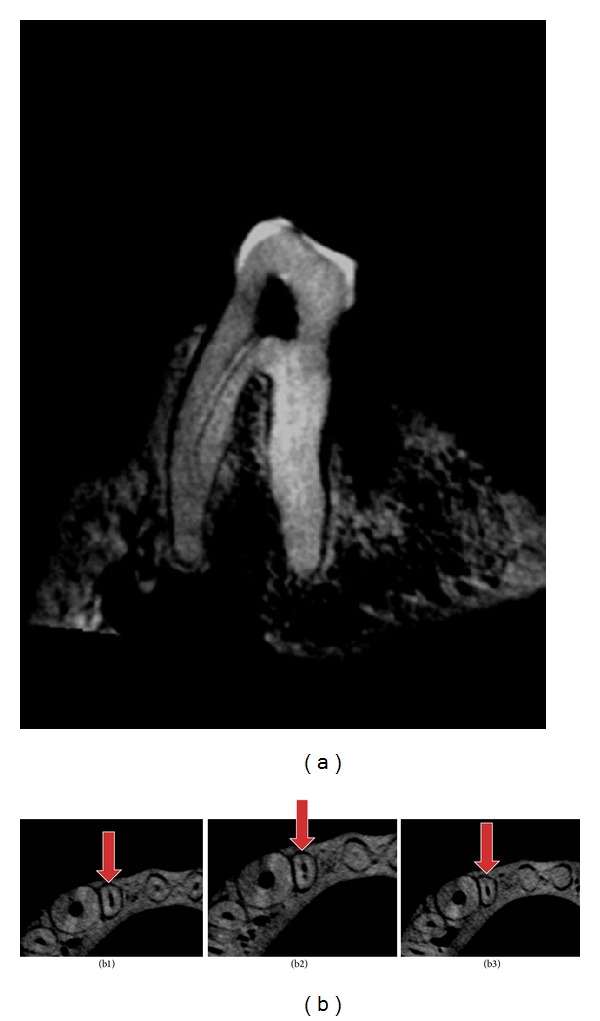
(a) Mesial root of the lower left second premolar of the Sumatran orangutan showing two root canals with corresponding two foramina; (b1, b2, and b3) a series of axial views of the lower lateral incisor (shown by arrow) of Bornean orangutan showing type III canal morphology of Vertucci's classification, where the canal begins as one, branches into 2 in the middle, and unites to become one.

The nonmetrical parameters explored the number of canals per root, number of foramina detected in each root, and Vertucci's classification of root canal variations. In general, all the teeth have one canal with one apical foramen per root. However, one exception was noticed in the mesial root of the lower left second premolar of the Sumatran orangutan, where there were two root canals with corresponding two foramina (Figure 5(a)). Unfortunately only the left second premolar was available for scanning as the right corresponding tooth was missing. Apart from this variation it was noticed that Bornean orangutan showed type III Vertucci's classification in the lower lateral incisors (Figure 5(b1–b3)).

## 4. Discussion

Orangutans, coming from the family of great apes and being native of the rainforests of Borneo and Sumatra, have many similarities as well as differences in their dentition with humans. The most unique feature observed among the two orangutans from this study was the number of roots in the first and second premolar in both jaws. Maxillary first and second premolars have three roots, while the mandible premolars have only two. Among human beings, the maxillary first premolar has two roots and the second premolar has only one, while in the mandibular jaw, the first and second premolar are with only one root [19]. It is also interesting to note the difference in root canal morphology in orangutans as compared to human dentition. In human beings, 64.4% of the mandibular first molars have two canals [20]. This was however not noticed in both the orangutans, where the mandibular first molar showed only one canal in each root. Moreover, the Sumatran orangutan exhibited two canals in the mesial root of the mandibular second premolar, unlike the Bornean orangutan. This difference could possibly be due to variations between the two species or maybe even a gender difference.

The average human being maxillary central incisor length is 23.5 mm, with a crown length of 10.5 mm and root length of 13 mm [20]. In comparison, the maxillary central incisors of the orangutans were significantly longer, whereby the length of the Bornean and Sumatran orangutans was 35.6 and 34.6 mm, respectively, with larger enamel covered surface. This could be explained due to the variation in the diet pattern, where the orangutan would need stronger anteriors and thicker enamel for gouging barks of trees.

In ape dentitions, the common molar size sequence is assumed to be M_3_ > M_2_ > M_1_, and this sequence is held in contrast to the assumed human sequence, M_1_ > M_2_ > M_3_ [21]. In this study, it is evident that the teeth length values decreased from the first premolar to the third molar in both the arches. This is quite typical in human beings where the 1st tooth of each class of teeth is the least variable and exhibits the strongest characteristics observed in that class of teeth, with the exception of the lower anterior incisors. It is noticed that even in the Bornean orangutan the lower lateral incisors were indeed longer than the central incisor, a finding similar to humans and as reported by Gingerich and Schoeninger [22].

The molars exhibited a root canal length between 11 and 19.5 mm. The large variations in their length are due to calcification of the roots as both the orangutans could have died at an advanced age. Similar to human the most conspicuous change is the decreasing volume of the pulp chamber and root canal brought about by continuous secondary dentine formation. In old teeth, the root canal is often no more than a thin channel and in certain occasions appears almost obliterated completely [23]. This is also evident in these orangutans where the continuous constrictions of the pulpal cavity cause reduction of the vascular supply to the pulp, thereby causing age related changes on the tooth. Furthermore, some of the root canals of the Sumatran orangutan teeth were partially calcified while some were completely obliterated (Figure 4). This indicated that the animal was probably at an advanced age when it died. It is estimated [24] that orangutans can live more than 40 years in the wild and between 50 and 60 years in captivity. Another similarity between orangutan and human was in length of the palatal root in the maxillary molars where the palatal root was longer than mesiobuccal and distobuccal roots with even the palatal root in the maxillary premolars following the same pattern.

Veterinary dentists must be aware that there are many pathways the root canals take to reach the apex. The pulp canal system may be complex and canals may branch, divide, and rejoin. Caution must however be expressed as it is difficult to determine the number of canals in each root employing either the coronal or sagittal images as there were superimposition of these canals [25, 26]. In these cases, a search employing the axial view becomes useful. The lower lateral incisor of the Bornean orangutan in this study showed type III Vertucci's classification which was surprisingly unique (Figures 5(b1–b3)).

## 5. Conclusions

Variations in the morphology of teeth amongst the orangutans have both genetic and environmental influences. This information may be of considerable value in studies of primate evolution and in assessing their biological affinities and even their migratory patterns. This study also has provided some guidelines with regard to the root canal system in orangutan for dental treatment, particularly during endodontic therapy. It is therefore necessary to have proper radiological or imaging investigations prior to any dental treatment of an orangutan.

## Figures and Tables

**Table 1:** Distribution of the orangutan teeth employed in this study.

Orangutan		I_1_	I_2_	C	PM_1_	PM_2_	M_1_	M_2_	M_3_	Total
Bornean	Max.	2	2	2	2	2	2	2	2	16
	Mand.	2	2	2	2	2	2	2	2	16

Sumatran	Max.	2	2	2	2	2	0	0	0	10
	Mand.	0	2	2	2	1	1	0	0	8

Max.: maxillary; Mand.: mandibular; I_1_: central incisor; I_2_: lateral incisor; C: canine; PM_1_: first premolar; PM_2_: second premolar; M_1_: first molar; M_2_: second molar; M_3_: third molar.

**Table 2:** Anterior teeth measurements.

		Bornean orangutan				Sumatran orangutan	
		TL	ECCH	LPS	TL	ECCH	LPS
Max. I_1_	R	35.6	12.2	32.1	34.6	11.3	31
	L	35.3	13.8	31.9	33.6	10.5	29.8
Max. I_2_	R	29.2	7.1	24.8	30.6	8.6	25.4
	L	29.9	8.4	25.5	30.9	8.4	26.5
Max. C	R	39.9	8.9	34.5	45.7	8.1	38.2
	L	39.6	8.9	34.6	43.7	8.2	37.7

Mand. I_1_	R	31.2	10.8	27.9	ms	ms	ms
	L	32.2	11.2	26.9	ms	ms	ms
Mand. I_2_	R	33.1	9.8	28.6	33.8	7.5	29.7
	L	33.6	10.5	29.2	32.9	7.7	31.7
Mand. C	R	42.7	12.8	39.4	42.9	10.4	38.3
	L	44.9	13.3	40.9	45.4	12.2	40.6

TL: tooth length; ECCH: enamel covered crown height; LPS: length of the pulp space; R: right; L: left; ms: missing tooth.

**Table:** (a)

		Bornean orangutan						Sumatran orangutan					
		TL	CL	ECCH	RL			TL	CL	ECCH	RL		
					MB	DB	P				MB	DB	P
Max. PM_1_	R	28.3	11.3	7.5	16.9	14.2	18.7	26	8.7	6.1	17.1	14.7	17.2
	L	29.1	11.4	9	18.1	14.3	19.2	25.9	7.2	5.9	18.8	18.4	21.5
Max. PM_2_	R	23.3	9.3	5.4	13.5	11.4	15	ms	ms	ms	ms	ms	ms
	L	24.8	10.1	6.3	15	13.4	16	23.2	7.3	4.7	16	14.7	17.7
Max. M_1_	R	22	7.5	4.8	13.9	14.5	16.2	24.2	7.9	5.3	16	14.6	18.3
	L	22.4	7.3	5.2	14.7	14.7	17	ms	ms	ms	ms	ms	ms
Max. M_2_	R	18.9	7.3	3.5	11.4	12.1	15	ms	ms	ms	ms	ms	ms
	L	19	7.9	3.1	11.1	11.2	14.8	ms	ms	ms	ms	ms	ms
Max. M_3_	R	17.6	6.4	4.4	11.1	11.6	11.9	ms	ms	ms	ms	ms	ms
	L	16.8	6.5	3.1	10.7	11	11.1	ms	ms	ms	ms	ms	ms

**Table:** (b)

		Bornean orangutan					Sumatran orangutan				
		TL	CL	ECCH	RL		TL	CL	ECCH	RL	
					M	D				M	D
Mand. PM_1_	R	31.1	10.4	7.7	20.8	18.1	30.2	8.2	5.3	22.2	20.5
	L	29.9	8.8	5.4	21	18.2	32.2	7.5	7.2	23.8	19.6
Mand. PM_2_	R	26.8	8.1	5.3	19	19	ms	ms	ms	ms	ms
	L	26.1	7.6	4.8	18.5	18.5	27.8	8.4	4.5	17.7	16.9
Mand. M_1_	R	24.1	5.4	4	18.3	16.8	25.5	6.5	4.6	18.2	17.4
	L	24.2	6.2	4	18.9	17.6	ms	ms	ms	ms	ms
Mand. M_2_	R	22.3	6.8	3.9	15.8	15	ms	ms	ms	ms	ms
	L	21.8	7	3.3	14.8	14.3	ms	ms	ms	ms	ms
Mand. M_3_	R	17.9	7.7	5.1	11.3	11.3	ms	ms	ms	ms	ms
	L	20	7.6	4.2	12.1	13	ms	ms	ms	ms	ms

R: right; L: left; TL: tooth length; CL: Crown length; ECCH: enamel covered area; LPS: length of the pulp space; RL: root length; MB: mesiobuccal root; DB: distobuccal root; P: palatal root; M: mesial root; DB: distal root; ms: missing tooth.

**Table:** (a)

		Bornean orangutan												Sumatran orangutan											
		Root canal length			Root canal width									Root canal length			Root canal width								
		MB	DB	P	MB			DB			P			MB	DB	P	MB			DB			P		
					co	m	ap	co	m	ap	co	m	ap				co	m	ap	co	m	ap	co	m	ap
Max. PM_1_	R	17	14	19	1.1	0.5	0.4	0.6	0.4	0.3	1	1	0.5	cal.	cal.	cal.	cal.	cal.	cal.	cal.	cal.	cal.	*cal.*	*cal.*	*cal.*
	L	18	14	19	0.9	0.5	0.3	0.8	0.7	0.6	3	0.7	0.9	cal.	cal.	cal.	cal.	cal.	cal.	cal.	cal.	cal.	*cal.*	*cal. *	*cal. *
Max. PM_2_	R	14	13	15	1	0.5	0.3	0.5	0.4	0.3	1.6	1	0.8	ms	ms	ms	ms	ms	ms	ms	ms	ms	ms	ms	ms
	L	16	14	18	1.2	0.5	0.6	0.4	0.4	0.4	2.3	0.7	0.9	cal.	cal.	cal.	cal.	cal.	cal.	cal.	cal.	cal.	cal.	cal.	cal.
Max. M_1_	R	14	15	16	1.1	0.4	0.6	0.7	0.4	0.5	2	0.7	0.7	cal.	cal.	cal.	cal.	cal.	cal.	cal.	cal.	cal.	cal.	cal.	cal.
	L	15	15	17	0.9	0.5	0.2	0.5	0.3	0.1	2	0.7	0.7	ms	ms	ms	ms	ms	ms	ms	ms	ms	ms	ms	ms
Max. M_2_	R	12	12	14	1	0.5	0.4	0.5	0.4	0.4	2.1	0.8	0.8	ms	ms	ms	ms	ms	ms	ms	ms	ms	ms	ms	ms
	L	11	12	14	1	0.5	0.4	0.4	0.5	0.3	2.4	0.9	1.2	ms	ms	ms	ms	ms	ms	ms	ms	ms	ms	ms	ms
Max. M_3_	R	12	11	12	1.1	0.6	0.6	0.3	0.3	0.2	1.6	0.6	0.6	ms	ms	ms	ms	ms	ms	ms	ms	ms	ms	ms	ms
	L	12	11	11	0.6	0.7	0.3	0.2	0.5	0.3	1.8	1.2	0.6	ms	ms	ms	ms	ms	ms	ms	ms	ms	ms	ms	ms

**Table:** (b)

		Bornean orangutan								Sumatran orangutan							
		Root canal length		Root canal width						Root canal length		Root canal width					
		M	D	M			D			M	D	M			D		
				co	m	ap	co	m	ap			co	m	ap	co	m	ap
Mand. PM_1_	R	21	19	1.3	0.7	0.6	0.7	0.6	0.4	cal.	19	cal.	cal.	cal.	1.5	0.5	0.6
	L	21	19	0.7	0.4	0.6	0.8	0.6	0.4	25	21	1.8	1.1	0.6	2	0.7	0.7
Mand. PM_2_	R	19	19	1.1	0.6	0.5	1.1	0.8	0.3	ms	ms	ms	ms	ms	ms	ms	ms
	L	19	18	0.9	0.3	0.3	0.8	0.3	0.2	18	ca	1.5	0.3	0.2	cal.	cal.	cal.
Mand. M_1_	R	19	18	1.9	0.4	0.5	1.8	0.7	0.6	19	19	1.5	0.6	0.5	1.3	0.4	0.5
	L	20	18	1.8	0.5	0.4	1.5	0.5	0.5	ms	ms	ms	ms	ms	ms	ms	ms
Mand. M_2_	R	16	15	2.1	0.7	0.6	2.5	1	0.6	ms	ms	ms	ms	ms	ms	ms	ms
	L	15	14	1.5	0.4	0.3	1.6	0.7	0.6	ms	ms	ms	ms	ms	ms	ms	ms
Mand. M_3_	R	13	12	1.7	1.2	0.7	1	1.2	0.9	ms	ms	ms	ms	ms	ms	ms	ms
	L	12	14	1.8	0.8	0.5	0.9	0.7	0.7	ms	ms	ms	ms	ms	ms	ms	ms

R: right; L: left; MB: mesiobuccal root; DB: distobuccal root; P: palatal root; M: mesial root; D: distal root; co: canal orifice; m: midpoint between orifice and apex; ap: apex; ms: missing tooth; *cal.*: partially calcified root canal; cal.: fully calcified root canal.
